# The wriggly resilience of eels to angler catch-and-release

**DOI:** 10.1093/conphys/coaa050

**Published:** 2020-05-23

**Authors:** Madeleine S Killacky

**Affiliations:** Centre for Arthurian Studies, Bangor University, Bangor, Gwynedd, Wales LL57 2DG, UK

Catch-and-release angling is a hugely popular sport, but what happens to non-targeted species (i.e. bycatch) after they are released? Interestingly, American eels seem to be quite resilient following an angling interaction. In fact, it turns out that there is little difference in the outcome between cutting the line and removing the hook when releasing them. Like with all good research, however, more needs to be done—especially in real-life field settings—to know for sure.

Every year, young American eels (*Anguilla rostrata*) make the famously secretive and long journey (~3000 km) from the salty gyre of the Sargasso Sea into the coastal, estuarine and fresh waters of North America. Eels are catadromous fishes, meaning that they hatch in salt water but live out their adult lives in mostly fresh water before returning to the Sargasso Sea to spawn and die. American eels are an ‘endangered’ species and so anglers do not target eels; however, sometimes eels end up as bycatch. Therefore, understanding the effects of angling on released eels is important for the conservation management of this fish.

Until recently, no studies had examined the resilience of American eels following an angling event and so Margaret Litt and her team decided to do just that. They investigated the effects of having a shallow or deeply imbedded hook and whether or not releasing the eel by cutting the line or removing the hook entirely had an effect on the eel's immediate survival.

Litt and her team captured 207 eels and separated them into four groups: control, sham (placebo), shallow-hooked and deep-hooked. The control group remained in the holding tank and the others were anaesthetized using clove oil. This method of anaesthetization allowed the researchers to carefully insert the hooks, as eels are otherwise very difficult to handle. Clove oil is a good choice because it wears off quickly and has little to no lasting effects on fishes. Hooked eels were removed from the tank by the line, and the line was then either cut or the hook was manually removed.

Interestingly, no eels died during the 7-day post-angling period, and there was little or no injury to the eels. Although eels that had the hooks manually removed spent a longer time out of the water, neither method posed a significant threat to these eels over the short term.

These eels, therefore, defied expectations that they would experience significant adverse effects following a simulated angling interaction. Maybe this makes sense, though. Eels need to be hardy and resilient creatures to make the long and dangerous journey from the Sargasso Sea and back again. They can survive out of water for extended periods of time, and they can comfortably live in a variety of salt and fresh water environments. Therefore, it may not be surprising that they can survive catch-and-release too. Although Litt and her team found that incidental capture by anglers does not pose a major threat to the American eel if released properly, more research in a field setting needs to be performed in order to continue monitoring and conserving this very special and endangered fish.

**Illustrations:** Erin Walsh, ewalsh.sci@gmail.com


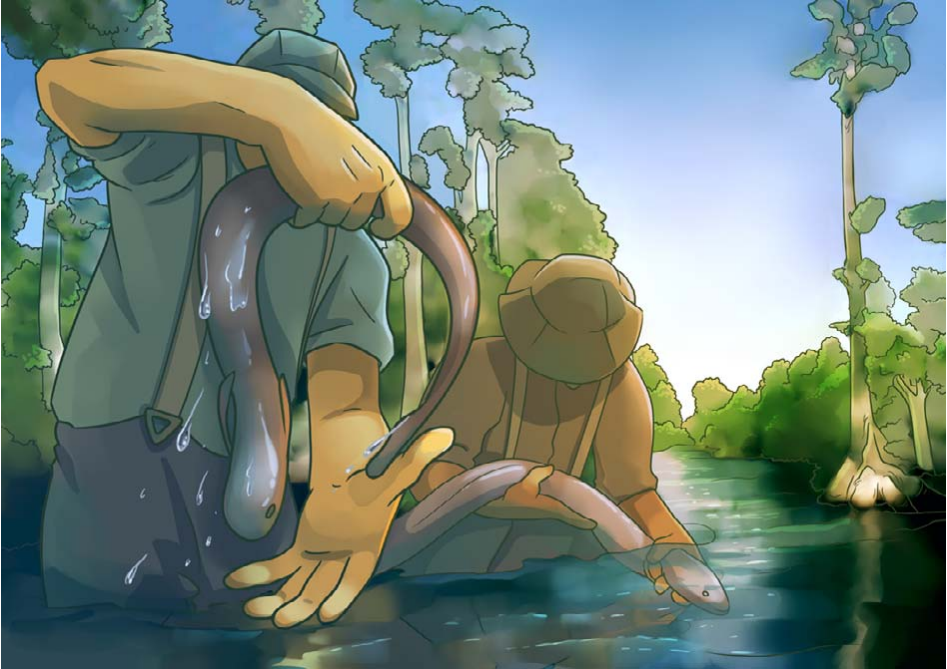


**Editor:** Jodie L. Rummer
